# Influence of Nurses’ Perfectionism on Professional Quality of Life: The Role of Submissive Compassion

**DOI:** 10.3390/healthcare14162539

**Published:** 2026-08-13

**Authors:** Yoshikazu Noda, Moa Yokota, Kumiko Kishimoto, Kenichi Asano

**Affiliations:** 1 Research Center for Child Mental Development, Chiba University, 1-8-1 Inohana, Chuo-ku, Chiba 268-0856, Japan; 2 Department of Nursing, Faculty of Human Care at Makuhari, Tohto University, 1-1 Hibino, Mihama-ku, Chiba 261-0021, Japan; 3 Nerima Child Guidance Center, 5-28-3 Toyotamakita, Nerima-ku, Tokyo 176-0012, Japan; 4 Faculty of Nursing, Musashino University, 3-3-3 Ariake, Koto-ku, Tokyo 135-8181, Japan; 5 Institute of Human Sciences, University of Tsukuba, 1-1-1 Tennodai, Tsukuba 305-8572, Japan

**Keywords:** perfectionism, submissive compassion, secondary traumatic stress, burnout, nurses, ProQOL

## Abstract

**Background/Objectives**: Nurses are frequently exposed to emotionally demanding situations, placing them at risk of adverse psychological outcomes such as secondary traumatic stress (STS) and burnout. Personality traits, particularly perfectionism, may influence these outcomes. Submissive compassion, characterized by caring behavior motivated by a desire for approval or avoidance of rejection, may play an explanatory role in these relationships. **Methods**: This study examined whether submissive compassion was statistically associated with the relationship between perfectionism and professional quality of life (ProQOL) among hospital nurses, while accounting for potential confounding and moderating factors. A cross-sectional online survey was conducted among ward nurses working in hospitals in Japan. Participants completed measures of perfectionism, submissive compassion, and ProQOL. Data from 218 nurses were analyzed using structural equation modeling. **Results**: Socially prescribed perfectionism was positively associated with submissive compassion and STS. Submissive compassion was significantly associated with the indirect relationship between socially prescribed perfectionism and STS. Age moderated the association between submissive compassion and STS, with stronger indirect effects observed among older nurses. Additionally, self-oriented perfectionism showed a relationship with stress-related outcomes, whereas socially prescribed perfectionism was associated with burnout. **Conclusions**: The findings suggest that submissive compassion may contribute to the association between perfectionism and adverse psychological outcomes among nurses. These results underscore the potential importance of maladaptive compassion motives in understanding how perfectionism relates to STS in this population and may help inform interventions aimed at supporting nurses’ psychological well-being.

## 1. Introduction

Among professionals in helping occupations—whose primary role is to improve the physical, mental, or social well-being of others—some individuals experience maladaptive psychological states resulting from providing care, which ultimately lead to a decline in their quality of life (QOL). Stamm et al. [1] proposed the concept of professional quality of life (ProQOL), which encompasses burnout, secondary traumatic stress (STS), and compassion satisfaction (CS), all of which play a significant role in shaping helpers’ well-being. Providing care to individuals who have experienced trauma can have a particularly strong impact on helping professionals’ QOL [1]. Nurses, in particular, are required to engage directly and continuously with patients facing life-threatening situations, making them especially vulnerable to STS and burnout [2]. STS refers to rapid stress reactions resulting from indirect exposure to trauma [3], characterized by symptoms such as emotional exhaustion, intrusive thoughts related to patients’ traumatic experiences, and difficulty separating professional from personal life, similar to posttraumatic stress. Burnout, as defined by Maslach et al. [4], is a syndrome commonly observed among individuals working in people-oriented professions, characterized primarily by emotional exhaustion and cynicism. As individuals’ emotional resources become depleted through work, they experience a diminished capacity to engage with others at a psychological level (e.g., impatient listening, monotone responses, or a tendency to engage only in surface-level conversations). This depletion often leads to negative and detached attitudes toward clients. Furthermore, previous research has shown that higher levels of STS and burnout are associated with increased turnover intention among nurses [5]. These two constructs were previously conceptualized together as compassion fatigue, defined as a state of stress arising from deep involvement with and exposure to the suffering or trauma of those receiving care [6]. Nurses are particularly susceptible to compassion fatigue because of the emotional demands of responding empathically to patients’ psychological needs [7]. In addition to these emotional challenges, nurses frequently work in demanding healthcare environments characterized by heavy workloads, workplace bullying, and exposure to workplace violence [8,9,10]. Such working conditions may adversely affect nurses’ well-being and professional functioning, contributing to workforce instability and persistent nursing shortages. Against this backdrop, understanding factors associated with nurses’ ProQOL has become increasingly important for improving both healthcare organizations and patient care outcomes.

Given the negative consequences of STS and burnout—including increased turnover, reduced quality of care, and both tangible (e.g., financial costs associated with staff replacement) and intangible (e.g., decreased morale and organizational cohesion) organizational losses [11]—researchers have sought to identify their predictors. This issue is particularly important in Japan, where nursing workforce shortages remain a significant challenge. Therefore, identifying psychological factors associated with nurses’ ProQOL may help inform strategies to support nurses’ well-being and promote workforce retention. One important intrapersonal factor is perfectionism, defined as a tendency toward excessively critical self-evaluation and setting unrealistically high standards [12]. Perfectionism is considered a relatively stable personality trait that shapes consistent patterns of cognition and behavior [13]. Individuals with high levels of perfectionism are more likely to experience psychological distress, and they may also be more vulnerable to STS and burnout [14]. Furthermore, the concept of submissive compassion may help explain how caregiving motivations influence ProQOL outcomes. Submissive compassion refers to the tendency to help others to gain approval or avoid rejection [15]. Unlike authentic compassion, which involves empathic concern and a genuine motivation to alleviate suffering [15], submissive compassion is driven by self-evaluative concerns. It is associated with negative self-evaluation and reduced self-reassurance and is predicted by factors such as social vulnerability, fear of inadequacy as a caregiver, and self-image goals [15,16].

Nurses may be particularly prone to submissive compassion because of both professional expectations and personal tendencies. They are often expected to embody the ideal image of healthcare professionals who are consistently compassionate and self-sacrificing [17], and may adapt their behavior to meet these expectations [18]. This professional pressure can increase their sensitivity to external evaluations, leading nurses to engage in caregiving behaviors motivated by others’ perceptions. Accordingly, these tendencies may also be associated with perfectionism. Nurses with perfectionistic tendencies may experience higher levels of job stress and workaholism [19]. One relevant dimension is socially prescribed perfectionism (SPP), which involves striving to meet perceived external standards. Simultaneously, self-oriented perfectionism (SOP)—reflecting internalized ideals—may also promote caregiving driven by the desire to fulfill one’s own standards [20]. Both SPP and SOP may reinforce submissive compassion, as caregiving becomes motivated by meeting external or internal standards rather than responding authentically to others’ needs. Higher levels of submissive compassion have been shown to interfere with genuine empathic engagement [16]. Consequently, nurses with high levels of submissive compassion may struggle to provide effective support, leading to reduced CS and increased vulnerability to STS and burnout.

The present study is also informed by Conservation of Resources (COR) theory [21,22]. COR theory posits that individuals are motivated to acquire, maintain, and protect valued personal and social resources and that psychological distress occurs when these resources are threatened or depleted. From this perspective, perfectionistic tendencies may function as strategies to protect self-related resources such as self-worth, competence, and social approval. Similarly, submissive compassion may serve as an interpersonal strategy intended to preserve relational resources, including acceptance, belongingness, and positive evaluation by others. Although these strategies may initially help individuals avoid the perceived loss of resources, their chronic use may require substantial emotional investment and ultimately contribute to resource depletion, thereby increasing vulnerability to burnout and STS, while reducing CS. In particular, perfectionistic concerns—especially socially prescribed perfectionism—may increase individuals’ efforts to obtain and maintain social approval. Such efforts may manifest as submissive compassion, which requires sustained emotional investment in interpersonal relationships. Over time, this investment may contribute to resource depletion, increasing vulnerability to secondary traumatic stress and burnout while reducing compassion satisfaction. Thus, COR theory provides a useful framework for understanding the proposed relationships among perfectionism, submissive compassion, and ProQOL among nurses.

Although several factors associated with nurses’ ProQOL have been described, research gaps remain. Particularly, while previous studies have examined perfectionism and ProQOL separately, little is known about whether submissive compassion plays a role in the relationship between perfectionistic tendencies and ProQOL among nurses. Furthermore, studies conducted in Japan have reported that psychological characteristics, including perfectionistic tendencies, are associated with nurses’ occupational adaptation and work-related well-being [23,24]. However, the relationships among perfectionism, submissive compassion, and ProQOL have not been examined simultaneously within the Japanese nursing context. Addressing this gap may contribute to a more comprehensive understanding of the factors associated with nurses’ ProQOL and help inform culturally relevant interventions. The present study examined a conceptual model in which SOP and SPP contribute to higher levels of submissive compassion, which in turn increases STS and burnout while decreasing CS. Specifically, this study investigated the pattern of associations among perfectionistic tendencies, submissive compassion, and dimensions of ProQOL among Japanese nurses to clarify factors associated with nurses’ ProQOL.

## 2. Materials and Methods

### 2.1. Study Aim, Design, and Setting

In this survey study, we aimed to examine the relationships among perfectionism, submissive compassion, and ProQOL among nurses using path analysis while adjusting for relevant confounding factors. Specifically, we hypothesized that:

**H1.**  *Self-oriented perfectionism would be directly associated with compassion satisfaction and indirectly associated with compassion satisfaction through pathways involving submissive compassion.*

**H2.**  *Self-oriented perfectionism would be directly associated with secondary traumatic stress and indirectly associated with secondary traumatic stress through pathways involving submissive compassion.*

**H3.**  *Self-oriented perfectionism would be directly associated with burnout and indirectly associated with burnout through pathways involving submissive compassion.*

**H4.**  *Socially prescribed perfectionism would be directly associated with compassion satisfaction and indirectly associated with compassion satisfaction through pathways involving submissive compassion.*

**H5.**  *Socially prescribed perfectionism would be directly associated with secondary traumatic stress and indirectly associated with secondary traumatic stress through pathways involving submissive compassion.*

**H6.**  *Socially prescribed perfectionism would be directly associated with burnout and indirectly associated with burnout through pathways involving submissive compassion.*

**Figure 1:**
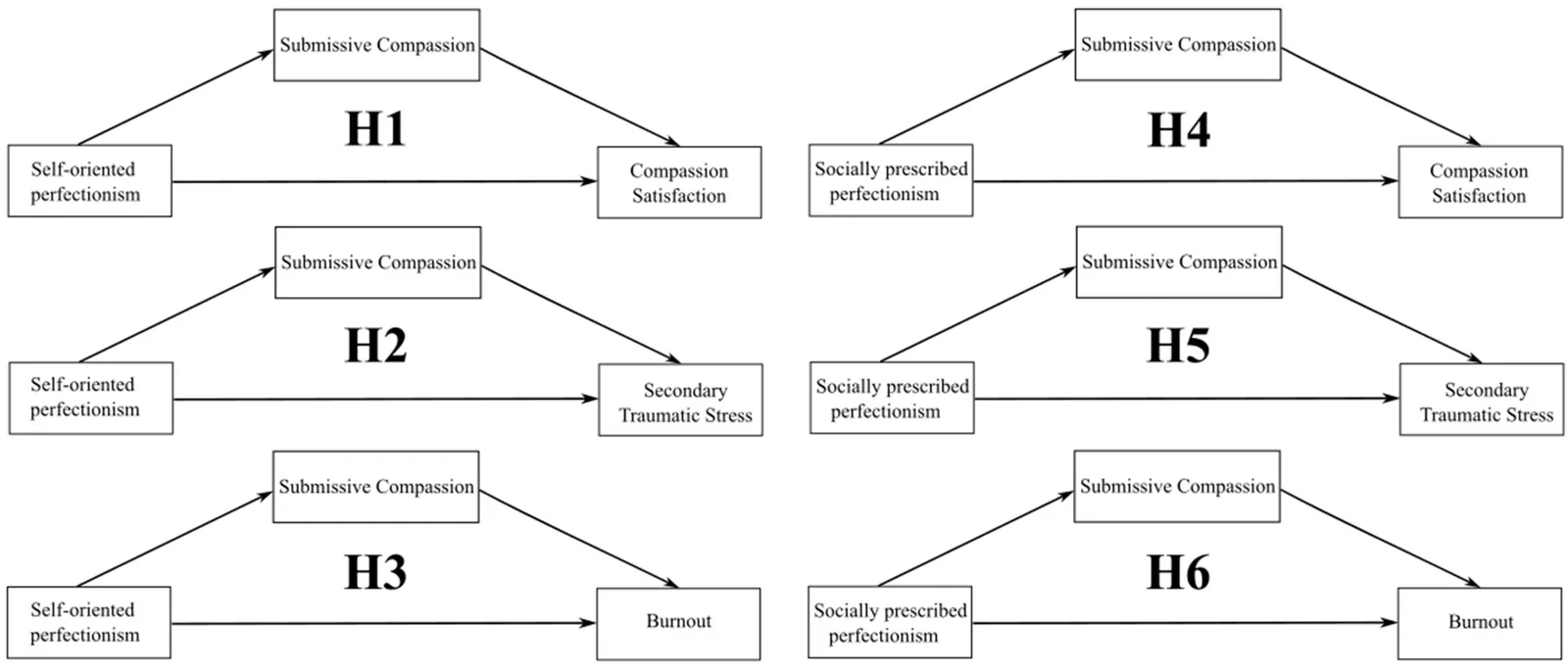
Hypothesized path model illustrating the associations between the multidimensional perfectionism scale and professional quality of life through submissive compassion.

Figure 1 presents the hypothesized paths. Furthermore, the moderating role of age was explored in an additional analysis and was not specified as an a priori hypothesis.

### 2.2. Procedure

We conducted a cross-sectional survey of ward nurses working in hospitals across Japan using an online research system. Participants were recruited through Freeasy, a commercial web-based survey platform operated by iBRIDGE Inc. (Osaka, Japan). The questionnaire was developed by the research team and administered via the platform’s self-service survey system. Registered panel members were screened for eligibility, and individuals who identified themselves as hospital nurses were invited to participate. Data collection continued until the predetermined target sample size was reached. Because the survey was administered through an existing online panel, information regarding the number of panel members who received the invitation was unavailable from the provider, preventing the calculation of a response rate. Consequently, the representativeness of the sample and the potential influence of selection and nonresponse bias could not be fully evaluated and should therefore be considered when interpreting the findings. The participants were nurses aged 23–68 years. The initial target sample size was set at more than 200 participants, based on previous studies with similar designs [25] and commonly accepted recommendations for path analysis and structural equation modeling, which suggest that a sample size of approximately 200 is generally adequate for stable parameter estimation [26]. Given the complexity of the proposed path model and the number of estimated parameters, a sample size exceeding 200 was considered sufficient to ensure reliable parameter estimation and model evaluation. A priori power analysis was not conducted because the study aimed to evaluate an overall path model rather than test a single effect size; however, a post hoc Monte Carlo simulation was performed to evaluate statistical power for the final model. In total, 226 participants completed the survey. Data cleaning was subsequently conducted, and data from 218 participants were included in the analysis after excluding those who provided identical responses to all items on scales containing reverse-coded questions. All subsequent analyses were performed using this final sample. Participant recruitment began in February 2024 and concluded in March 2024. Ethical approval was granted by the Clinical Research Ethics Committee of Mejiro University on 23 November 2023 (approval number: 23human-013). Informed consent was obtained electronically. All participants provided written informed consent via e-mail.

### 2.3. Measurement

#### 2.3.1. Multidimensional Perfectionism Scale

We used the Japanese version of the Multidimensional Perfectionism Scale (MPS) to assess perfectionism [27]. The Japanese MPS is a translated and validated version of the original MPS developed by Hewitt and Flett [28]. It is among the most widely used measures of perfectionism, and its Japanese version has also been commonly used to assess perfectionistic tendencies in Japan. The scale comprises three factors: SOP, other-oriented perfectionism (OOP), and SPP. In the present study, we focused on SOP and SPP. In a previous study, the Cronbach’s alpha values of the Japanese MPS coefficients were reported as follows: SOP = 0.83 and SPP = 0.65 [1], with the latter demonstrating relatively lower internal consistency. Because previous validations of the Japanese MPS have primarily relied on scale scores rather than confirmatory factor analytic approaches, the factorial structure of the SOP and SPP subscales was re-examined in the present sample. To obtain a clearer and more interpretable factor structure, items with low factor loadings (<0.40) or substantial cross-loadings were removed in accordance with established psychometric recommendations. Confirmatory factor analysis (CFA) was conducted to verify whether the hypothesized two-factor structure was supported when only these subscales were administered. Participants were instructed to indicate how well each statement applied to them by selecting their responses on a 7-point Likert scale ranging from 1 (not at all applicable) to 7 (very applicable).

#### 2.3.2. Submissive Compassion Scale

We used the Japanese version of the Submissive Compassion Scale [29] to assess submissive compassion. The scale was developed based on the Submissive Compassion Scale originally proposed by Catarino et al. [15] and consists of 15 items representing a single factor of submissive compassion. Responses were rated on a 5-point Likert scale ranging from 0 (not at all like me) to 4 (extremely like me). The Cronbach’s alpha of the Japanese Submissive Compassion Scale (0.92) indicated good internal consistency [29]. Because the Japanese version of the Submissive Compassion Scale has not yet been published in a peer-reviewed validation study, findings involving this construct should be interpreted with caution and in light of the scale’s current stage of development.

#### 2.3.3. Japanese Version of the ProQOL Scale for Nurses

The ProQOL, developed by Stamm et al. [1], has been used worldwide to measure the positive and negative effects of working with individuals who have experienced stressful or traumatic events, comprising the three subscales: CS, STS, and burnout. The ProQOL has been translated into Finnish, French, German, Hebrew, Italian, Spanish, Croatian, Portuguese, and Russian [1]. The Japanese Version of the ProQOL Scale for Nurses was developed by Fukumori et al. [30]. Participants responded to each item using a 5-point Likert scale: 1 (never), 2 (rarely), 3 (sometimes), 4 (often), 5 (very often). All scores were reported as T-scores. A previous paper reported the following Cronbach’s alpha values: STS = 0.84, CS = 0.89, and burnout = 0.76 [30].

#### 2.3.4. Confounding and Moderating Factors

The potential confounders considered in the analysis were age, sex, marital status, length of employment, and educational attainment. Age and length of employment were treated as continuous variables. Sex, educational attainment, and marital status were coded as dichotomous variables. Educational attainment was categorized as high school graduation versus completion of postsecondary education (college, technical school, or university). Marital status was classified as either unmarried or married. As these variables could influence the relationships among the primary variables of interest, they were included as covariates when they did not demonstrate a moderating effect.

### 2.4. Analyses

Data analysis proceeded in four stages. First, CFA was performed to examine whether the two-factor structure of SOP and SPP was supported and to evaluate the adequacy of the factor structure [31]. Items with low factor loadings (standardized loadings < 0.40) and those showing substantial cross-loadings on both factors were removed to improve the clarity and interpretability of the factor structure, consistent with commonly accepted criteria for confirmatory factor analysis. Loadings of 0.40 or greater were considered acceptable for item retention. Descriptive statistics were computed, and the distribution of each measure was examined using probability–probability plots and Shapiro–Wilk tests. Partial correlations were calculated to examine the associations among the study variables. Additionally, multicollinearity was evaluated using variance inflation factors. Internal consistency reliability was assessed using Cronbach’s alpha; coefficients greater than 0.7 were considered indicative of satisfactory reliability [32,33]. Second, linear regression analyses were conducted to examine the associations among subscale scores of the MPS, Submissive Compassion Scale, and ProQOL using bias-corrected bootstrapping confidence intervals based on 5000 resampling replicates. Assumptions of linearity, homoscedasticity, and residual normality were evaluated using residual diagnostics. Residual normality was assessed through visual inspection of histograms and normal Q–Q plots of the residuals. Homoscedasticity was evaluated by plotting residuals against fitted values, which demonstrated no substantial evidence of heteroscedasticity. Although some observed variables deviated from normality, the regression residuals did not exhibit substantial deviations from normality. Furthermore, the use of bias-corrected bootstrapping reduced the influence of potential violations of distributional assumptions. Third, path analysis with 5000 bias-corrected bootstrap resamples was conducted to explore the relationships among the study variables. Although the conceptual model was derived from previous literature and COR theory, these analyses were exploratory in nature and conducted to examine the extent to which the theoretically proposed pathways were supported in the present sample. Fourth, moderation and moderated path analyses were conducted to examine whether the selected demographic variables moderated the associations of interest. Variables that did not exhibit moderation effects were treated as covariates. To evaluate the practical significance of the age moderation effect, Cohen’s f^2^ was calculated based on the change in explained variance attributable to the interaction term. The effect size was estimated using the difference in R^2^ values between the model including the interaction term and the corresponding model without the interaction term, using the formula: f^2^ = (R^2^full − R^2^reduced)/(1 − R^2^full). Cohen’s benchmarks of 0.02, 0.15, and 0.35 indicated small, medium, and large effects, respectively.

Model adequacy was assessed using the comparative fit index (CFI), Tucker–Lewis index (TLI), and root mean square error of approximation (RMSEA). Following commonly used recommendations, CFI and TLI values of 0.90 or higher and RMSEA values of 0.08 or lower were considered indicative of acceptable model fit, whereas CFI and TLI values of 0.95 or higher indicated good model fit [34,35]. Because the unconditional observed-variable path model was saturated (i.e., just-identified: df = 0), fit indices were uninformative for evaluating that model. Therefore, interpretation of the unconditional model focused on parameter estimates, indirect effects, and bootstrap confidence intervals. Conversely, model fit indices were used to evaluate the moderated path model, which had positive degrees of freedom. To reduce the risk of inflated Type I errors arising from multiple hypothesis testing involving the MPS, Submissive Compassion Scale, and a ProQOL, Bonferroni correction was applied across 11 regression tests. Accordingly, statistical significance was determined using an adjusted significance threshold of 0.0045 (0.05/11). To evaluate the adequacy of the sample size, a post hoc Monte Carlo simulation was conducted based on the observed parameter estimates from the final model. Statistical power for detecting the indirect effect was estimated using 5000 replications and the observed sample size. Structural equation modeling, path analysis, moderation analyses, and the post hoc Monte Carlo simulation were performed using Mplus version 9.0 (Muthén & Muthén, Los Angeles, CA, USA). All other statistical analyses were performed using Stata version 18 (StataCorp LLC., College Station, TX, USA).

## 3. Results

### 3.1. Participant Characteristics

Table 1 presents the characteristics of the 218 participants who completed the questionnaires. Participants’ ages ranged from 23 to 68 years, and 85% of the participants were female. Approximately 62% were married, and most participants (96.3%) had received postsecondary education. The assumption of normality was violated for some measures. Multicollinearity was not observed among the study variables.

### 3.2. Confirmatory Factor Analysis

Table S1 presents the results of CFA for the MPS. CFA was performed to examine whether the scale demonstrated a two-factor structure. The model fit indices were acceptable, supporting the results that confirmed a two-factor structure (RMSEA = 0.073, CFI = 0.918, and TLI = 0.903). Several items had factor loadings of <0.4 or cross-loadings on both factors; thus, a few items were removed (SOP: six items, 28, 32, 34, 36, 40, and 42; SPP: six items, 5, 9, 13, 21, 25, and 37).

### 3.3. Partial Correlation and Multiple Regression Analyses

SPP subscale scores on the MPS were positively correlated with submissive compassion and the STS and burnout subscale scores on the ProQOL, whereas the SOP subscale scores on the MPS were not significantly correlated with submissive compassion (Table S2).

To further examine the independent associations among perfectionism, submissive compassion, and the three dimensions of ProQOL while controlling for potential confounding variables, multiple regression analyses were conducted. Table 2 summarizes the regression models examining factors associated with submissive compassion, CS, STS, and burnout.

As shown in Table 2, SPP was significantly associated with submissive compassion, STS, and burnout, whereas SOP was significantly associated only with STS. In addition, submissive compassion was significantly associated with STS but not with CS or burnout.

Specifically, SPP subscale scores on the MPS were statistically associated with STS subscale scores on the ProQOL (SPP: β = 0.387, 95% CI = 0.292–0.482, *p* < 0.001). Submissive compassion scores were significantly associated with SPP (β = 0.284, 95% CI = 0.172–0.395, *p* < 0.001) and STS subscale scores on the ProQOL (β = 0.399, 95% CI = 0.275–0.522, *p* < 0.001). Multiple regression analysis results indicated that higher submissive compassion scores were associated with higher levels of SPP on the MPS and STS subscale scores on the ProQOL. Inspection of residual diagnostics indicated no substantial violations of normality, linearity, or homoscedasticity assumptions. Therefore, path analysis was subsequently conducted to further examine these relationships.

### 3.4. Unconditional Path Analysis with Confounding Factors

Table 3 presents the results of path analysis adjusted for confounding factors and conducted without moderation effects. The unconditional path model was saturated (just-identified: df = 0) and therefore yielded perfect fit indices (RMSEA = 0.000, CFI = 1.000, and TLI = 1.000). Because fit indices are not informative for just-identified models, the interpretation focused on the estimated path coefficients, indirect effects, bootstrap confidence intervals, and explained variance. The “A” pathway between SPP subscale scores on the MPS and submissive compassion scores was significant (β = 0.284, 95% CI = 0.172 to 0.395, *p* < 0.001). There was a significant association with the “B” pathway between submissive compassion scores and STS subscale scores on the ProQOL (β = 0.262, 95% CI = 0.158 to 0.366, *p* < 0.001). A direct effect revealed a significant association between SPP and STS subscale scores on the MPS (β = 0.313, 95% CI = 0.221 to 0.405, *p* < 0.001). The indirect and total effects resulted in the following coefficients: indirect effect: β = 0.074, 95% CI = 0.029 to 0.119, *p* < 0.01; total effect: β = 0.387, 95% CI = 0.292 to 0.482, *p* < 0.001. The R-square value for the models was 0.395, explaining 40% of the variance in STS subscale scores of the MPS. Results for the remaining models are shown in Table S3, and no significant indirect effects were observed, except for H5. No significant indirect effects through submissive compassion were observed for CS or burnout.

### 3.5. Moderation and Moderated Path Analysis

**Figure 2:**
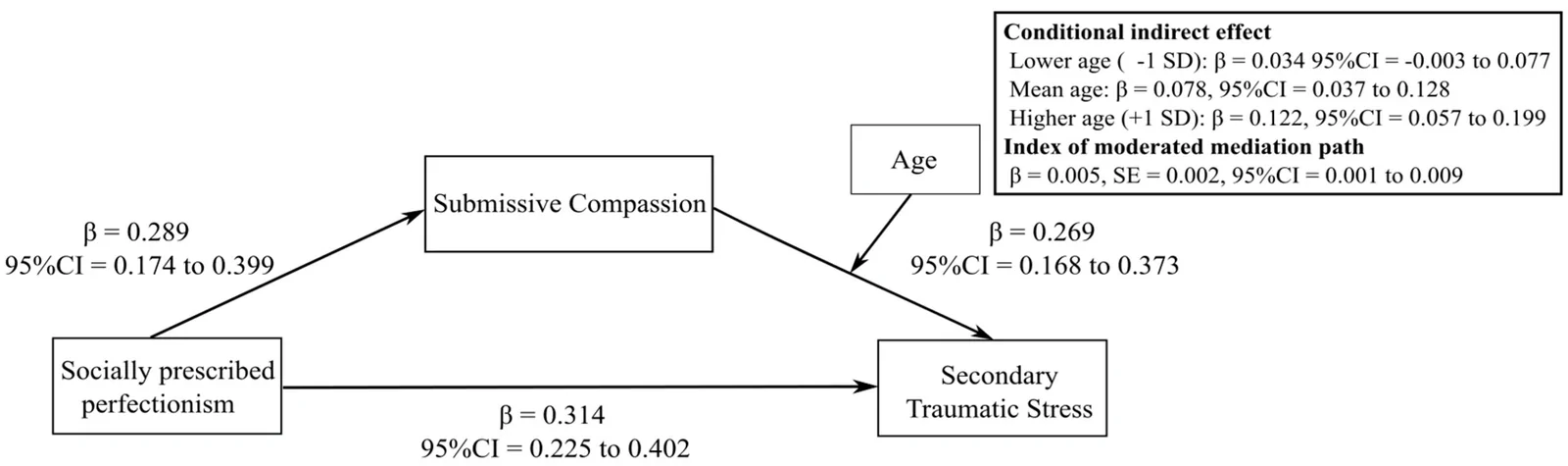
Final moderated path model of secondary traumatic stress among nurses (H5). Model fit indices: RMSEA = 0.039, CFI = 0.995, and TLI = 0.966.

To examine whether demographic characteristics moderated the proposed pathways, moderation analyses were conducted using age, sex, marital status, length of occupational experience, and educational level as covariates (data not shown in the table). The results indicated that the interaction between submissive compassion scores and age was statistically significant for STS (β = 0.016, 95% CI = 0.006 to 0.026, *p* < 0.001). Based on the moderation analysis results, Figure 2 illustrates the results of the moderated path analysis.

The conditional indirect effects at three levels of age were estimated as follows: lower (−1 SD; β = 0.034, 95% CI = −0.003 to 0.077), mean (β = 0.078, 95% CI = 0.037 to 0.128), and higher (+1 SD; β = 0.122, 95% CI = 0.057 to 0.199).

The estimated indirect effects increased across age levels and were consistent with the positive index of moderated mediation. The index of moderated mediation was positive and statistically significant (β = 0.005, standard error = 0.002, 95% CI = 0.001 to 0.009), suggesting that age modestly moderated the indirect association between SPP subscale scores and STS through submissive compassion. Additionally, all three pathways were statistically significant: path A (β = 0.289, 95% CI = 0.174 to 0.399), path B (β = 0.269, 95% CI = 0.168 to 0.373), and the direct effect (β = 0.314, 95% CI = 0.225 to 0.402). These estimates differ slightly from those reported in Table 3 because they were obtained from the moderated path model rather than the unconditional mediation model. However, the effect size was small (Cohen’s f^2^ = 0.031), suggesting that the practical significance of the moderation effect was limited. Therefore, the findings provide statistical support for the moderated path hypothesis, although the magnitude of the age-related moderation was modest. The model fit indices for the moderated path model indicated acceptable fit (RMSEA = 0.039, CFI = 0.995, and TLI = 0.966). Additionally, the χ^2^ test was not statistically significant (χ^2^ = 2.654, df = 2, *p* = 0.265).

### 3.6. Post Hoc Monte Carlo Simulation

A post hoc Monte Carlo simulation based on the observed model parameters was conducted to evaluate the statistical power to detect the indirect effect. The estimated power for detecting the indirect effect was 0.993.

## 4. Discussion

### 4.1. Main Findings

The present study aimed to examine whether submissive compassion was statistically associated with the relationship between the MPS and ProQOL among Japanese nurses working in hospital settings. The hypothesized association between SPP, submissive compassion, and STS was supported. Additionally, age moderated the association between submissive compassion and STS in the path model.

The present findings suggest that nurses with higher levels of SPP may be more vulnerable to STS, with submissive compassion representing one potential pathway in this association. This interpretation is consistent with previous research indicating that greater emotional involvement in patients’ suffering may increase vulnerability to STS and that submissive compassion may heighten such emotional involvement [2,36,37].

The findings are also broadly consistent with COR theory. From this perspective, both perfectionistic tendencies and submissive compassion may involve sustained efforts to protect valued self-related and interpersonal resources, such as social approval, acceptance, and self-worth. Although these efforts may initially serve a protective function, continued emotional investment may contribute to resource depletion over time. In emotionally demanding nursing environments, this process may increase vulnerability to STS.

However, the indirect effect accounted for only approximately 19% of the total effect, suggesting that submissive compassion represents only one potential pathway linking SPP with STS. Other psychological mechanisms, such as difficulties with emotion regulation [38] and resilience [39], may also contribute to this association and warrant further investigation.

Overall, the findings provide preliminary evidence that SPP may be associated with psychological burden in caregiving contexts, with submissive compassion representing one possible pathway. These findings may have practical implications for supporting nurses’ psychological well-being. Previous research has suggested that reducing the harmful effects of SPP may involve reducing external pressures, preventing these pressures from determining one’s sense of self-worth, and strengthening resilience and coping capacity [14]. In particular, mindfulness- and self-efficacy-based interventions have been shown to reduce perfectionism and improve self-efficacy, thereby mitigating stress and anxiety [40]. Furthermore, hospitals may consider providing psychological counseling and mental health support programs to help nurses cope with occupational stressors and maintain psychological well-being [41].

### 4.2. Moderated Effect: Age as a Moderator

The moderated path analysis indicated that age strengthened the indirect association between SPP and STS through submissive compassion. Although the age-related moderation was statistically significant, the magnitude of the moderation effect was small (Cohen’s f^2^ = 0.031). Consistent with this small effect size, the conditional indirect effects differed only modestly across age levels.

One possible explanation is that older nurses may have accumulated greater exposure to emotionally demanding clinical situations [42]. However, previous studies have reported inconsistent findings regarding the role of age as an influencing factor [42]. These inconsistent findings may be attributable to other factors, such as psychological resilience, which may influence the extent to which individuals experience STS. Previous research has demonstrated that higher psychological resilience is associated with lower levels of STS among nurses, suggesting that resilience may function as a protective factor that reduces stress responses following repeated exposure to patients’ suffering [43]. However, resilience was not directly assessed in the present study.

Therefore, the practical significance of the observed age-related moderation should be interpreted cautiously and requires replication in independent samples. Future research should consider examining resilience and other potentially relevant factors to better understand the conditions under which age may influence the association between submissive compassion and STS.

### 4.3. Other Findings

SOP and SPP were significantly associated with STS and burnout, respectively. The association between SOP and STS suggests that nurses reporting higher levels of SOP also tended to report higher levels of stress related to traumatic experiences encountered at work. In such situations, individuals may intensify their efforts to maintain high levels of performance and control, potentially increasing psychological burden when these standards are difficult to achieve in emotionally demanding caregiving contexts.

In contrast, SPP, which reflects perceived external expectations, was associated with burnout. Nurses who feel compelled to meet the expectations of patients, colleagues, or supervisors may experience chronic interpersonal and role-related stress, which may contribute to emotional exhaustion and reduced professional efficacy. Taken together, these findings suggest that different dimensions of perfectionism may be associated with different aspects of psychological well-being.

Interestingly, although submissive compassion was positively associated with STS, it was not significantly associated with CS. One possible explanation is that CS and STS represent distinct dimensions of ProQOL. Previous research has primarily linked submissive compassion to indicators of psychological distress, such as depression, anxiety, stress, and shame, rather than to positive well-being outcomes [16]. Thus, submissive compassion may be more closely related to psychological burden than to compassion satisfaction. Although submissive compassion may increase emotional burden, it may not necessarily diminish the sense of meaning or fulfillment derived from helping patients. This interpretation may help explain the non-significant association between submissive compassion and CS observed in the present study.

While the present study focused primarily on individual psychological factors, organizational factors may also contribute to the development of STS. Previous studies have suggested that excessive workload, inadequate supervision, limited organizational support, and high emotional demands in the workplace contribute to STS among helping professionals [2,42,44]. Therefore, interventions targeting only individual characteristics may be insufficient. In addition to individual-focused interventions, creating supportive organizational environments, promoting access to supervision and consultation, and implementing workplace policies that reduce chronic occupational stress may help mitigate the risk of STS. Future research should examine how individual vulnerabilities and organizational factors interact in the development of STS.

These findings highlight the importance of considering the multifaceted nature of perfectionism when examining its associations with nurses’ well-being in clinical settings.

### 4.4. Measurement Issues

As several items were removed during the factor analytic process, the resulting factor structure differed from that of the original MPS. Accordingly, the modified SOP and SPP subscales may not reflect the constructs in exactly the same manner as originally conceptualized by the scale developers. This potential alteration in construct representation should be considered when interpreting the findings, and direct comparisons with studies using the original MPS should be made with caution. The item-removal process was conducted within the same sample used to evaluate the measurement model; therefore, the possibility of sample-specific overfitting cannot be ruled out. Consequently, the modified factor structure should not be considered fully equivalent to the original MPS without further psychometric evaluation and cross-validation in independent samples.

Although the CFA results generally supported the modified factor structure, the model demonstrated acceptable rather than excellent fit (CFI = 0.918, TLI = 0.903). Thus, the factorial validity of the modified MPS should be interpreted with some caution. The substantially higher internal consistency observed for the revised SPP subscale (Cronbach’s α = 0.88) compared with that reported for the original Japanese version of the MPS (α = 0.65) should also be interpreted cautiously. One possible explanation is that the removal of items with weaker loadings resulted in a more homogeneous set of items, thereby increasing internal consistency. Additionally, differences in sample characteristics between the present study and the original validation study may have contributed to the higher reliability estimate.

While the increase in reliability may indicate improved internal coherence among the retained items, it does not necessarily imply improved construct validity. Notably, the original SOP and SPP subscales demonstrated acceptable internal consistency in the present sample (Cronbach’s α = 0.88 and 0.82, respectively), and reliability remained acceptable after item removal (Cronbach’s α = 0.87 and 0.88, respectively). However, the item-reduction process may have narrowed the conceptual scope of the original perfectionism dimensions and altered their content coverage. Therefore, the modified factor structure should not be considered fully equivalent to the original MPS, and further psychometric validation and cross-validation in independent samples are warranted.

### 4.5. Theoretical and Practical Implications

The present findings provide preliminary evidence that submissive compassion may be one pathway through which SPP is associated with STS among nurses. Although the findings are exploratory and do not support causal inference, they suggest the potential importance of caregiving motivations in understanding nurses’ ProQOL.

From a theoretical perspective, the findings contribute to the literature by suggesting that submissive compassion may be relevant to understanding the association between SPP and STS. The findings also highlight the potential relevance of interpersonal and self-regulatory processes in ProQOL among nurses. In addition, the findings are broadly consistent with COR theory, suggesting that efforts to maintain social approval and meet perceived external expectations may gradually deplete psychological resources and increase vulnerability to occupational stress.

From a practical perspective, the findings suggest that interventions targeting perfectionistic concerns and adaptive coping strategies may help support nurses’ psychological well-being. In addition, healthcare organizations may help mitigate stress-related outcomes by providing psychological support and fostering supportive work environments.

### 4.6. Limitations and Future Research

The present research has several limitations. First, the cross-sectional design precludes causal inferences regarding the relationships between perfectionism, submissive compassion, and ProQOL. Although the path analysis identified a significant indirect association between SPP and STS through submissive compassion, the directionality of these relationships cannot be conclusively determined. Longitudinal and experimental studies are required to clarify the underlying mechanisms.

Second, the analyses relied entirely on self-reported measures, which may have been subject to common method bias, social desirability effects, and recall bias. In particular, responses to items on compassion- and empathy-related constructs may be influenced by participants’ tendencies to present themselves in a socially desirable manner, especially among professional groups such as nurses. Because all variables were assessed using the same method and at the same time point, common method variance may have inflated the observed associations among the study variables. Future research would benefit from incorporating multiple data sources, such as supervisor ratings, peer evaluations, or longitudinal assessments, to reduce the potential influence of common method bias.

Third, because the sample consisted exclusively of Japanese nurses, the generalizability of the findings to other cultural and healthcare contexts may be limited. Although the post hoc Monte Carlo simulation suggested adequate power for detecting the observed indirect effect, an a priori sample size calculation was not conducted. Therefore, the adequacy of the sample size, particularly for the moderated path analyses, cannot be fully established. Accordingly, the age-stratified findings should be interpreted with caution and require replication in larger samples. Furthermore, because an a priori sample size calculation was not performed and the response rate could not be determined, the adequacy and representativeness of the sample should be interpreted cautiously, particularly for the moderated path analyses.

Fourth, although several demographic and professional characteristics were included as covariates and moderators, other potentially important individual and occupational factors were not assessed. These may include personality traits (e.g., neuroticism), clinical specialty, workload, nurse-to-patient ratio, organizational support, previous exposure to traumatic events, and psychological resilience [2,42,44,45]. Moreover, because participants were recruited through a commercial online survey panel, the total number of eligible and contacted nurses was unavailable, and a response rate could not be calculated. Consequently, the potential for selection bias and the representativeness of the sample could not be fully assessed. In addition, detailed information regarding their employing institutions, hospital types, and geographical locations was unavailable. Therefore, institutional and regional differences could not be examined, which may limit the generalizability of the findings. Residual confounding therefore cannot be ruled out, and future research should incorporate these variables to better clarify the relationships among perfectionism, submissive compassion, and STS.

Fifth, the measurement of perfectionism was limited to self-oriented and socially prescribed dimensions, excluding other relevant aspects such as OOP. Furthermore, several items from the original MPS were removed during the factor analytic process, resulting in a modified factor structure. Although the revised subscales demonstrated acceptable reliability, the removal of items may have altered the conceptual breadth and content coverage of the original perfectionism dimensions. Therefore, the findings should be interpreted with caution, as the modified scale structure may limit a comprehensive understanding of how different facets of perfectionism relate to caregiving and stress outcomes and may not be fully equivalent to the original MPS.

Sixth, the Japanese version of the Submissive Compassion Scale used in this study has not yet been described in an independent peer-reviewed validation study. Although preliminary psychometric evaluation has been conducted during the scale development process, evidence regarding construct validity, convergent and discriminant validity, test–retest reliability, predictive validity, measurement invariance, and replication across independent samples remains unavailable. Consequently, the associations and pathways involving submissive compassion identified in the present path analysis should be interpreted with caution. Therefore, the present findings should be regarded as hypothesis-generating rather than confirmatory evidence regarding the role of submissive compassion. Independent peer-reviewed validation of the Japanese Submissive Compassion Scale remains necessary before stronger conclusions regarding its mediating role can be drawn. Conclusions regarding the mediating role of submissive compassion are preliminary and require confirmation in future studies using independently validated measures.

Seventh, participants were recruited through a voluntary online research panel. Therefore, self-selection bias cannot be ruled out, as nurses who chose to participate may have differed systematically from those who did not participate. In addition, information regarding the number of panel members who received the survey invitation was unavailable from the survey provider; consequently, response rates could not be calculated, and potential nonresponse bias could not be evaluated. Furthermore, the representativeness of the sample relative to the broader population of Japanese hospital nurses could not be confirmed.

Finally, the unconditional path model was just-identified, and the moderated path model had only a small number of degrees of freedom. Therefore, conventional fit indices should be interpreted cautiously, as they may provide limited information regarding model adequacy. Additionally, since the final path model was derived from exploratory analyses, the findings should be interpreted with caution and validated in future studies using confirmatory analytic approaches.

A further limitation concerns the cultural and professional context of the study. The present sample consisted exclusively of Japanese nurses recruited through an online survey platform. Professional norms, emotional expression, interpersonal relationships, and perfectionistic tendencies may vary across cultural settings and healthcare systems. In particular, Japanese culture has been characterized as placing a relatively strong emphasis on social harmony, interpersonal sensitivity, and meeting others’ expectations, which may influence both SPP and submissive compassion. Therefore, caution is warranted when generalizing the present findings to healthcare professionals in other cultural contexts or countries with different healthcare environments. Future cross-cultural studies are needed to determine whether the observed relationships among perfectionism, submissive compassion, and STS are replicated across different healthcare systems and cultural settings.

Future research should employ longitudinal and confirmatory designs, examine additional individual and organizational determinants of STS, validate the modified MPS in independent samples, and investigate the generalizability of the findings across different cultural and healthcare settings.

## 5. Conclusions

This study suggests that submissive compassion is significantly associated with the relationship between SPP and STS among nurses. In addition, age modestly moderated this association, although the magnitude of the moderation effect was small. The present study suggests that maladaptive motivations underlying helping behaviors may be relevant to nurses’ ProQOL and psychological well-being. The findings also provide preliminary support for a COR-based interpretation, suggesting that efforts to maintain social approval and interpersonal acceptance may contribute to resource depletion in emotionally demanding nursing environments. From a practical perspective, interventions aimed at reducing excessive concern about others’ evaluations and promoting healthier forms of compassion may help support nurses in emotionally demanding healthcare environments. These findings should be interpreted with caution, however, given several methodological limitations, including the limited independent validation of the Submissive Compassion Scale, uncertainty regarding sample representativeness due to the unavailable response rate, and the absence of an a priori power analysis. Given the cross-sectional and exploratory nature of the study, future longitudinal research is needed to further examine these relationships and clarify their temporal ordering.

## Figures and Tables

**Table 1:** Demographic characteristics of the hospital nurses.

	N (%)	Mean	SD	Min	Max	Cronbach’s α
Age	218	41.8	9.63	23	68	
Sex						
Male	33 (15.1)					
Female	185 (84.9)					
Marital status						
Unmarried	83 (38.1)					
Married	135 (61.9)					
Occupational length	218	15.49	10.25	0	51	
Education						
High school	8 (3.7)					
College or more	210 (96.3)					
Multidimensional Perfectionism Scale						
Self-oriented perfectionism	218	39.74	9.48	13	63	0.87
Socially prescribed perfectionism	218	34.50	9.07	9	63	0.88
Submissive Compassion scale	218	23.62	7.47	10	49	0.91
ProQOL						
Compassion Satisfaction	218	30.82	7.20	10	47	0.90
Secondary Traumatic Stress	218	23.61	7.19	10	47	0.88
Burnout	218	28.18	5.55	12	44	0.72

Abbreviations: ProQOL, Professional Quality of Life Measure; SD, standard deviation.

**Table 2:** Linear regression analysis: Multidimensional Perfectionism Scale, submissive compassion, and ProQOL.

	Submissive Compassion				Compassion Satisfaction				Secondary Traumatic Stress				Burnout			
	*B*	SE	95% CI	*p*	*B*	SE	95% CI	*p*	*B*	SE	95% CI	*p*	*B*	SE	95% CI	*p*
Self-Oriented Perfectionism	0.075	0.056	−0.034 0.185		0.042	0.063	−0.083 0.166		**0.172**	**0.050**	**0.074 0.269**	**	0.077	0.044	−0.009 0.163	
Socially Prescribed Perfectionism	**0.284**	**0.057**	**0.172 0.395**	***	0.079	0.073	−0.063 0.221		**0.387**	**0.048**	**0.292 0.482**	***	**0.173**	**0.048**	**0.079 0.267**	***
Submissive Compassion					0.164	0.074	0.020 0.308		**0.399**	**0.063**	**0.275 0.522**	***	0.044	0.049	−0.053 0.140	

** *p* < 0.01; ***; *p* < 0.001. Bold fonts are statistically significant. *B*s are non-standardized coefficients. Bonferroni-corrected *p*-values are under 0.0045. Bootstrapped regression analysis using 5000 samples. The covariates are age, sex, education, marital status, and occupational duration. Abbreviations: ProQOL, Professional Quality of Life Measure; SE, standard error; CI, confidence interval.

**Table 3:** Unconditional path analysis: Multidimensional Perfectionism Scale, submissive compassion, and ProQOL.

Path	H5			
	*B*	SE	95% CI	*p*
A	**0.284**	**0.057**	**0.172 0.395**	***
B	**0.262**	**0.053**	**0.158 0.366**	***
Direct effect	**0.313**	**0.047**	**0.221 0.405**	***
Indirect effect	**0.074**	**0.023**	**0.029 0.119**	**
Total effect	**0.387**	**0.048**	**0.292 0.482**	***
R2	**0.395**	**0.055**		***

** *p* < 0.01; *** *p* < 0.001. Bold fonts are statistically significant. *B*s are non-standardized coefficients. Bias-corrected bootstrapping path analysis using 5000 samples. The covariates are age, sex, education, marital status, and occupational duration. Path A: Socially Prescribed Perfectionism to Secondary Traumatic Stress. Path B: Submissive Compassion to Secondary Traumatic Stress. Abbreviations: ProQOL, Professional Quality of Life Measure; SE, standard error; CI, confidence interval.

## Data Availability

The datasets generated and analyzed in the current study are available from the corresponding author upon reasonable request. However, the data are not publicly available due to the inclusion of information that could compromise the privacy of the research participants.

## Supplementary Materials

The following supporting information can be downloaded at: https://www.mdpi.com/article/10.3390/healthcare14162539/s1, Table S1: Factor loadings for the revised multidimensional perfectionism scale among Japanese nurses. Table S2: Partial correlations between measurements. Table S3: Unconditional path analysis: Multidimensional Perfectionism Scale, submissive compassion, and ProQOL.
