# Supplementary material for: Influence of Nurses’ Perfectionism on Professional Quality of Life: The Role of Submissive Compassion
Source: Healthcare (Basel). 2026 Aug 13;14(16):2539. doi: 10.3390/healthcare14162539 (PMC13512268; doi:10.3390/healthcare14162539)
Supplement: Supplementary file 1 [file healthcare-14-02539-s001.zip › healthcare-4375835-supplementary.pdf]

Table S1: Factor loadings for the revised multidimensional perfectionism scale among Japanese nurses

Items No.	Items	Factor loadings	SE
Self-oriented perfectionism			
1	When I am working on something, I cannot relax until it is perfect.	0.776	0.032
6	One of my goals is to be perfect in everything I do.	0.832	0.026
8	I never aim for perfection in my work.	0.445	0.058
12	I seldom feel the need to be perfect.	0.447	0.058
14	I strive to be as perfect as I can be.	0.862	0.023
15	It is very important that I am perfect in everything I attempt.	0.698	0.039
17	I strive to be the best at everything I do.	0.469	0.056
20	I demand nothing less than perfection from myself.	0.816	0.027
23	It makes me uneasy to see an error in my work.	0.442	0.058
Socially prescribed perfectionism			
11	The better I do, the better I am expected to do.	0.543	0.051
18	The people around me expect me to succeed at everything I do.	0.675	0.041
30	Others think I am okay, even when I do not succeed.	0.685	0.042
31	I feel that people are too demanding of me.	0.762	0.033
33	Although they may not show it, other people get very upset with me when I slip up.	0.667	0.042
35	My family expects me to be perfect.	0.466	0.058
39	People expect nothing less than perfection from me.	0.779	0.033
41	People expect more from me than I am capable of giving.	0.733	0.036
44	People around me think I am still competent even if I make a mistake.	0.689	0.040

Factor Loadings are standardized coefficients, with significance set at $P < .001$.

Model fit indices: root mean square error of approximation = 0.073; comparative fit index = 0.918; Tucker–Lewis index = 0.903

Abbreviations: SE, standard error; CI, confidence interval

Table S2: Partial correlations between measurements

	Self-oriented perfectionism	*p*	Socially prescribed perfectionism	*p*	Submissive compassion	*p*	Compassion satisfaction	*p*	Secondary traumatic stress	*p*	Burnout
Self-oriented perfectionism	1										
Socially prescribed perfectionism	0.47	***	1								
Submissive compassion	0.10		0.35	***	1						
Compassion satisfaction	0.06		0.10		0.22	**	1				
Secondary traumatic stress	0.23	***	0.51	***	0.38	***	0.05		1		
Burnout	0.13		0.29	***	0.22	**	-0.18	**	0.54	***	1

** p <.01; ***; p <.001

Bootstrapping correlations were obtained using 5,000 samples.

The covariates were age, sex, education, marital status, and occupational duration.

Table S3: Unconditional path analysis: Multidimensional Perfectionism Scale, submissive compassion, and ProQOL

	H1: SOP → submissive compassion → CS				H2: SOP → submissive compassion → STS				H3: SOP → submissive compassion → Burnout			
Path	*B*	SE	95%CI	*p*	*B*	SE	95%CI	*p*	*B*	SE	95%CI	*p*
A	0.075	0.056	-0.035 0.181		0.075	0.056	-0.035 0.181		0.075	0.056	-0.035 0.181	
B	**0.160**	**0.071**	**0.018 0.296**	*	**0.381**	**0.060**	**0.259 0.496**	***	0.034	0.048	-0.062 0.130	
Direct effect	0.030	0.062	-0.088 0.153		**0.143**	**0.043**	**0.057 0.226**	**	0.075	0.044	-0.011 0.160	
Indirect effect	0.012	0.012	-0.005 0.040		0.029	0.022	-0.013 0.074		0.003	0.005	-0.006 0.014	
Total effect	0.042	0.063	-0.079 0.171		**0.172**	**0.050**	**0.074 0.268**	**	0.077	0.044	-0.009 0.162	
R2	0.073	0.039			**0.295**	**0.055**		***	0.080			*
	H4: SPP → submissive compassion → CS				H5: SPP → submissive compassion → STS				H6: SPP → submissive compassion → Burnout			
Path	*B*	SE	95%CI	*p*	*B*	SE	95%CI	*p*	*B*	SE	95%CI	*p*
A	**0.284**	**0.057**	**0.171 0.391**	***	**0.284**	**0.057**	**0.172 0.395**	***	**0.284**	**0.057**	**0.171 0.391**	***
B	0.147	0.073	0.000 0.288	*	**0.262**	**0.053**	**0.158 0.366**	***	-0.037	0.048	-0.131 0.058	
Direct effect	0.037	0.072	-0.101 0.183		**0.313**	**0.047**	**0.221 0.405**	***	**0.183**	**0.050**	**0.083 0.278**	***
Indirect effect	0.042	0.022	0.000 0.086		**0.074**	**0.023**	**0.029 0.119**	**	-0.010	0.014	-0.038 0.017	
Total effect	0.079	0.073	-0.062 0.225		**0.387**	**0.048**	**0.292 0.482**	***	**0.173**	**0.048**	**0.078 0.266**	***
R2	0.073	0.040			**0.395**	**0.055**		***	0.142	0.047		**

* $p < .05$; ** $p < .01$; *** $p < .001$

Bold fonts are statistically significant.

*B*s are non-standardized coefficients.

Bias-corrected bootstrapping path analysis was performed using 5,000 bootstrap samples.

The covariates were age, sex, education, marital status, and occupational duration.

Abbreviation: ProQOL, Professional Quality of Life Measure; SOP, Self-oriented perfectionism; SPP, Socially prescribed perfectionism; CS, compassion satisfaction; STS, Secondary traumatic stress; SD, standard error; CI, confidence interval
